# Study of risk factor of urinary calculi according to the association between stone composition with urine component

**DOI:** 10.1038/s41598-021-87733-7

**Published:** 2021-04-22

**Authors:** Pan Wang, Hongxian Zhang, Jiansuo Zhou, Shangjia Jin, Chong Liu, Boxin Yang, Liyan Cui

**Affiliations:** 1 Department of Laboratory Medicine, Peking University Third Hospital, Beijing, 100191 China; 2 Department of Urology, Peking University Third Hospital, Beijing, 100191 China

**Keywords:** Risk factors, Predictive markers

## Abstract

Urolithiasis is a common urinary disease with high recurrence. The risk factor for the recurrence of calculi is not very clear. The object of the present study was to evaluate the association between calculi composition and urine component and analyse the risk factor for the recurrence of urolithiasis. In this study, a total of 223 patients with calculi and healthy control were enrolled, and the components of the calculi and urina sanguinis collected before surgery were analysed. Of the 223 patients, 157 were males and 66 were females. According to the stone composition, the case group was subdivided into three groups. 129 patients had single calcium oxalate stones, 72 had calcium oxalate stones mixed with other stones and 22 had other type of stones excluding calcium oxalate stones. Urine biochemicals were analysed and the associations were found between the chemicals in each group. Multivariate logistic analysis demonstrated that reduced urinary magnesium and uric oxalic acid were independent risk factors when comparing all cases with normal controls. Only decreased urinary magnesium was found to be a risk factor comparing the single calcium oxalate group with normal control group. Low level of urinary magnesium and uric oxalic acid were found to be risk factors comparing the mixed calcium oxalate group with normal control group. No risk factor was found comparing the other stone group with normal control group. In conclusion, there were clear relationships between stone components and urine chemicals. Urine chemicals might be risk factors to predicate the occurrence of urolithiasis.

## Introduction

Urolithiasis is a common urinary disease that is characterized by the presence of stones within the renal pelvis, ureter, bladder or urethra^1^. Recent epidemiological data demonstrate that the prevalence and incidence rates of urolithiasis have increased considerably in nearly all countries^2–4^. According to the National Health and Nutrition Examination Survey, the prevalence of urolithiasis in the United States was 8.8% in 2010 compared with 3.2% in 1980^5^. In Asia, a stone-forming belt has stretched across western Asia and South Asia, including South Korea and Japan, with prevalence rates ranging from 5 to 19.1%^6,7^. In addition, the prevalence of kidney stones has also risen from 4 to 6.4% over the past 30 years in China owing to lifestyle and dietary changes^8^. Unfortunately, recurrence is common, with an estimated 5-year recurrence rate of up to 30–50%. Several studies demonstrated that the recurrence rate of urinary calculi in cured patients increased annually after the initial stone event^9–11^. These suggest that calculi formation, as a lifelong disease, should not only be treated but also prevented^12^.

Urinary stones usually consist of more than one substance^13^. Generally, the majority (80%) of urinary stones are composed of calcium oxalate mixed with calcium phosphate, followed by uric acid, struvite and cystine, accounting for approximately 9%, 10% and 1%, respectively^14^. Calcium oxalate stones mainly comprise two major constituents, calcium oxalate monohydrate and calcium oxalate dehydrate and frequently contain a mixture of the two substances. Excessive supersaturation of minerals in urine can lead to crystal formation, growth, aggregation and retention in the urinary tract. In addition, studies have demonstrated that the compositions of calculi have undergone substantial changes, and the incidences of calcium oxalate and calcium phosphate in calculi have increased^15^. However, the knowledge related to changes is still scarce.

Recent years great advances have been made in the treatment and surgical management of patients with urinary stones. Stones can be dispersed by shockwave lithotripsy to enable them to pass through the urethra or removed with the application of a series of minimally invasive methods, such as ureteroscopic lithotripsy and percutaneous nephrolithotripsy (PCNL). Besides, important progresses have also been made in understanding the calculi pathogenesis. Epidemiological studies have shown that calculi in the urinary system are not caused by a single factor but by the interactions of multiple factors, such as sex, ethnicity, age, climate, occupation and obesity^16–19^. Therefore, it is essential to identify potential risk factors for the formation of an initial calculus to provide a sound basis to prevent recurrence.

Urine contains multiple kinds of minerals and urine biomarkers to predicate and diagnose diseases at early stage has been widely accepted^20^. The supersaturation of minerals and a decrease in crystal inhibitors may increase the likelihood of stone formation, so analysing the composition of urine to discover the risks for stone formation might be a practical and reliable method. With this information in mind, we recruited 223 patients with calculi and some healthy control, stones and urina sanguinis were collected, then the mineralogical compositions of calculi as well as urinary oxalic acid, urinary citric acid, urinary uric acid and urinary phosphorus, urinary magnesium, and urinary calcium in calculi patients and normal control group were examined. Finally, the correlations between stone compositions with urine component were analysed and risk factors for the occurrence of urolithiasis were discovered and those might provide deeper insight into the aetiology of urolithiasis and improve its diagnosis and treatment.

## Materials and methods

### Patient selection

In this study, inpatients who diagnosed with urolithiasis underwent PCNL or ureteroscopic lithotripsy in the department of urology of the Peking University Third Hospital from November 2017 to April 2019 were enrolled. The purpose of the experiment was to examine the components of calculi and the morning urine and analyse the association between them. A total of 257 patients with confirmed stone component analysis were enrolled according to the enrolment conditions. Stone specimens were excluded from the study when records of patient age, sex, or urine composition before surgery (n = 34) were not available. In addition, 20 healthy persons who reported no nephropathy or renal and urogenital disorders were recruited. We identifies the Ethics Committee of Peking University Third Hospital approved the experiments, including any relevant details and confirms that all experiments were performed in accordance with relevant named guidelines and regulations. In addition, written informed consents were obtained from all the participants or their legal guardians.

### Stone analysis

Calculi specimens were washed with distilled water and then placed in an 80 °C oven for drying. A small portion (1 mg) of the stone specimen was mixed with 200 mg of potassium bromide, placed into an agate mortar, ground to a powder, and then pressed onto a translucent sheet with a thickness of approximately 1 mm for analysis by a LIIR-20 calculus infrared spectrometry automatic analyser (manufactured by Lambda Scientific Corporation, China).

### Urine components analysis

5 ml of the first morning urines were collected from healthy control and the inpatients before surgery and stored at – 80 °C until experimental use. Then, the components of urinary oxalic acid, urinary citric acid, urinary uric acid and urinary phosphorus, urinary magnesium, and urinary calcium were determined by the improved immunoturbidimetric method with an A25 automatic specific protein analyser.

### Statistical analysis

All statistical analyses were conducted using IBM SPSS, vision 23. Chi-squared tests were used to calculate the differences between groups. Person’s correlation test was used to analyse the correlation between stone compositions with urine components after log-data transformation. For comparison with the control group, logistic multifactor analyses were performed. The significance level was defined as statistically significant at *P* < 0.05 and very significant at *P* < 0.01.

## Results

### Characteristics of the population

Of the 223 patients in the study, the mean (standard deviation) age was 52.9 years (14.43 years), ranging from 19 to 86 years. The results demonstrated a sex predominance, with males accounting for 70.40% of the population and a male-to-female ratio of 2.38:1, which coincides with the epidemiological results^21^. The other detailed information of the patients and the control group is shown in Table 1. The characteristics of the control group matched well with those of the patient group.

**Table 1:** Population data.

Stone group			Normal control group	
Item	Number	Percentage (%)	Number	Percentage (%)
**Sex**				
Male	157	70.40	13	65.00
Female	66	29.60	7	35.00
**Age**				
< 45	65	29.15	2	10.00
45–60	68	30.49	9	45.00
> 60	90	40.36	9	45.00
**Stone location**				
Upper urinary trace	198	88.79		
Lower urinary tract	20	8.97		
Both upper and lower urinary tract	5	2.24		
**Group**				
Single COX group	129	57.85		
Mixed COX group	72	32.29		
Other group	22	9.87		

*COX* calcium oxalate.

### Stone components

Stones were classified according to their fundamental component. For example, in the single calcium oxalate group, there were 129 patients with only a calcium oxalate calculus. The mixed calcium oxalate group included 72 patients with stones containing calcium oxalate and carbonate apatite; calcium oxalate and anhydrous uric acid; calcium oxalate and magnesium ammonium phosphate; calcium oxalate and calcium hydrogen phosphate; calcium oxalate and l-cystine; or calcium oxalate, carbonate apatite and magnesium ammonium phosphate. The other stone group included 22 patients with a stone containing only anhydrous uric acid, only carbonate apatite, only l-cystine, only ammonium urate, or sodium urate and ammonium urate. There were no significant differences in the sex distributions among the three stone groups. The urine components of the patient groups and normal control group were examined, and the median and mean values of urinary oxalic acid, urinary citric acid, urinary uric acid, urinary phosphorus, urinary magnesium and urinary calcium for each group are shown in Table 2.

**Table 2:** The median and mean value of urinary components in each group.

Component	Single COX group	Mixed COX group	Other group	Normal control
**UUA (μmol/l)**				
Median	925.5	1005.05	801.05	1917.7
Mean	1113.279 ± 872.480	1102.251 ± 779.023	886.964 ± 751.801	1917.89 ± 163.18
**UP (mmol/l)**				
Median	5.5	4.465	8.3	14.815
Mean	9.545 ± 11.453	7.918 ± 9.002	8.949 ± 7.412	15.037 ± 1.347
**UMg (mmol/l)**				
Median	0.9	0.7	0.95	2.90
Mean	1.19 ± 1.021	1.036 ± 0.850	1.2 ± 0.899	2.865 ± 0.262
**UCA (mmol/l)**				
Median	1.1	1.2	0.9	2.50
Mean	1.605 ± 1.486	1.549 ± 1.365	1.214 ± 1.112	3.055 ± 0.529
**UOXA (mg/l)**				
Median	7.99	8.2	9.92	16.965
Mean	11.376 ± 12.760	10.9022 ± 9.908	14.909 ± 17.432	17.028 ± 1.382
**UCIT (mmol/l)**				
Median	1.3	1.515	2.08	6.39
Mean	3.297 ± 6.126	3.6408 ± 5.174	7.052 ± 12.319	6.452 ± 0.552

*UUA* urinary uric acid, *UP* urinary phosphorus, *UMg* urinary magnesium, *UCA* urinary calcium, *UOXA* uric oxalic acid, *UCIT* urinary citric acid.

### Correlation analysis of stone components with urine chemicals

**Figure 1:**
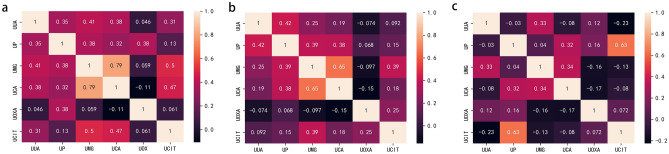
Association between stone components and urine chemicals were conducted in the single calcium oxalate group (**a**), mixed calcium oxalate group (**b**) and other stone group (**c**), respectively. *P* values were shown in the chart. The figure was performed using Python software version 3.8 (https://www.python.org/).

An analysis of calculi in patients with only calcium oxalate stone was conducted, and we found that urinary uric acid was positively correlated with urinary phosphorus, urinary magnesium, urinary calcium and urine citrate; urinary phosphorus was positively correlated with urinary magnesium, urinary calcium and uric acid; urinary magnesium was positively correlated with urinary calcium and urine citric acid; and urinary calcium was positively correlated with urinary citric acid (Fig. 1a). Next, the correlations were analysed in patients with mixed calcium oxalate stones. The results showed that urinary uric acid was positively correlated with urinary phosphorus and urinary magnesium; urinary phosphorus was positively correlated with urinary magnesium and urinary calcium; and urinary magnesium was positively correlated with urinary calcium and urine citric acid (Fig. 1b). Moreover, there was a positive correlation between only urinary phosphorus and urinary citrate in the analysis of calculi in patients with other stones (Fig. 1c).

### Multivariate logistic analysis of the risk factors

**Figure 2:**
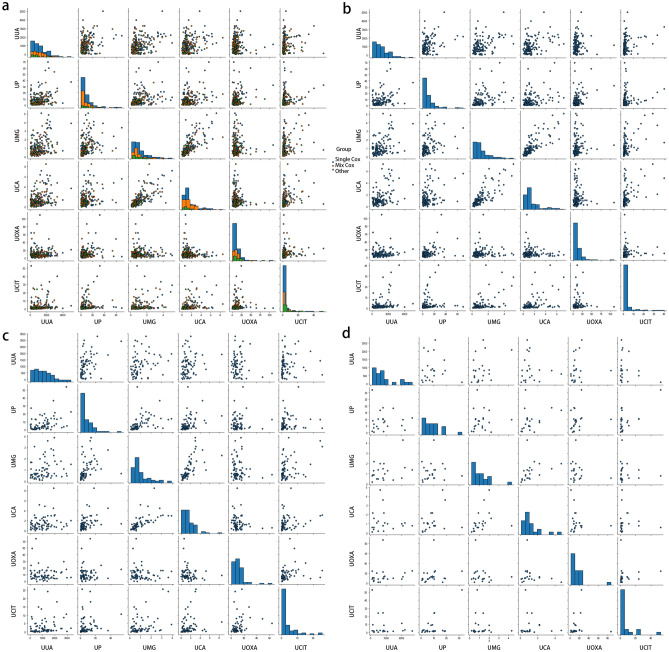
Risk factors were analysed through the multivariate logistic analysis between normal controls with all cases (**a**) or the single calcium oxalate group (**b**) or mixed calcium oxalate group (**c**) or other stone group (**d**), respectively. The figure was performed using Python software version 3.8 (https://www.python.org/).

In each group, the presence or absence of calculi (1 = yes, 0 = no) was considered the dependent variable, and urinary uric acid, urinary phosphorus, urinary magnesium, urinary calcium, urinary oxalic acid, and urinary citric acid were considered the independent variables for the multifactor logistic regression analysis. Decreased urinary magnesium and uric oxalic acid were independent risk factors in the multivariate logistic analysis of all cases and normal controls (Fig. 2a and Supplementary Table S1). Only reduced urinary magnesium was found to be a risk factor in the multivariate analysis of the single calcium oxalate group and normal control group (Fig. 2b and Supplementary Table S2). Decreased urinary magnesium and uric oxalic acid were shown as risk factors in the multivariate analysis in the mixed calcium oxalate group and normal control group (Fig. 2c and Supplementary Table S3). No risk factors were identified in the multivariate logistic analysis in the other stone group and normal control group (Fig. 2d and Supplementary Table S4).

## Discussion

It is well known that the supersaturation of urinary salts or a reduction in crystal inhibitors leads to an increased risk of stone formation; therefore, urine component testing can predict the risk of urinary stone formation in a timely matter and appropriate precautions can be taken to reduce the possibility of stone formation. In recent years, several studies have confirmed that most patients with urolithiasis suffer from hyperuric calcium, hyperuricuria, low urinary magnesium or low citric aciduria^22–26^. Twenty-four-hour urine biochemistry is generally considered the gold standard for the evaluation of urinary calculi metabolism, but some scholars have found that urine biochemical parameters of urina sanguinis are in good agreement with those of 24-h urine with respect to urine protein/urinary creatinine, urine phosphorus and uric acid, so urina sanguinis could be used as a substitute for 24-h urine for urinary calculi metabolism evaluation^27^. Our study analysed the first morning urine composition of calculus patients and normal people to reveal the relationships between urinary calcium, magnesium, phosphorus, oxalic acid, uric acid, citric acid and stone composition. The limitation of our experiment was that we did not compare and rectify the urine chemical data to confirm the consistency between morning urine with 24-h urine.

Most patients with urinary stones in China have calcium-containing stones, of which calcium oxalate stones are the primary type. Studies demonstrated that 90% of calcium oxalate dihydrate stones were associated with hypercalciuria^28^; however, other studies found that a high calcium diet was not related to the formation of hypercalciuria, and there was no correlation between dietary calcium and stone formation in adult men aged 60 years or older^29^. In addition, Sorensen et al.^30^ discovered that an increase in calcium in the diet can effectively reduce the incidence of kidney stones in female patients with urolithiasis. In this study, there was a positive linear correlation between urinary calcium and urinary uric acid, urinary magnesium, urinary phosphorus, and urinary citric acid in the single calcium oxalate stone group and with urinary phosphorus and urinary magnesium in the mixed calcium oxalate group.

The results of this study showed that the amount of urine oxalic acid in the total case group and mixed calcium oxalate group were also different from those in the normal control group in the multivariate analysis. After the multivariate logistic analysis, we found that every additional unit of urinary oxalic acid decreased the incidence of stones by 3.5% in the total case group and by 8% in the mixed calcium oxalate group. Several other reports^31,32^ manifest that hyperoxaluria is one of the independent risk factors for stone formation. In our experiment, we collected the morning urine from the inpatients who has been diagnosed with urolithiasis and ready for treatment, so the oxalic acid might have bound to calcium to form calcium oxalate. Therefore, the amount of the oxalic acid in cases group seems lower than control.

In recent years, the role of urinary magnesium in urolithiasis has been controversial^33^. Many studies have shown that urinary magnesium can inhibit the formation of calcium oxalate crystals and stones^34^, but some scholars discovered that the level of magnesium was higher in hypercalciuric stone formers than healthy subjects^35^. In our study, by comparing the correlations of urine stone components in each stone group, we found that in the single calcium oxalate stone group and the mixed calcium oxalate stone group, urinary magnesium was associated with urinary uric acid, urinary calcium, urinary phosphorus and urinary citric acid. Besides, the results of this study showed that the amount of urine magnesium was significantly different between the normal control group and the single calcium oxalate group and the mixed calcium oxalate group. After the multivariate logistic analysis, we found that similar to urine oxalic acid, in the case group, a 1 unit decrease in urine magnesium increased the risk of stone formation by 1.1 times, while in the single calcium oxalate group, the risk increased by 1.2 times; the risk increased as high as 1.5 times in the mixed calcium oxalate stone group. Given that 90% percent of the enrolled patients have calcium oxalate, our results coincided with above results that hypercalciuric stone formers had lower urinary magnesium.

Studies have shown that urease-producing bacteria in urinary tract infections can decompose ammonia in the urine and alkalize the urine, prolonged infection can promote the formation of calcium phosphate stones^36,37^. In this study, urinary phosphorus was associated with urinary uric acid, urinary magnesium, urinary calcium, and urinary oxalic acid in the single calcium oxalate stone group; with urinary uric acid, urinary magnesium, urinary calcium in the mixed calcium oxalate stone group; and with urinary oxalic acid in the other stone group.

In a study by Wang Shu et al.^17^, it was found that the stone composition in most patients in China contained calcium oxalate monohydrate or calcium oxalate dihydrate, and the correlation between urinary oxalic acid and stone formation was not obvious, consistent with the results of this study. In this study, urinary oxalic acid was associated with urinary phosphorus in the single calcium oxalate stone group; with urinary citric acid in the mixed calcium oxalate stone group. Numerous studies have shown that urinary citric acid can effectively inhibit the formation of urinary stones, and low citric acid in urine is a symptom of urolithiasis in some patients^38^. However, in this study, urine citric acid was positively correlated with a variety of elements that promote the formation of urinary stones, which is inconsistent with other reported results, possibly due to the small sample size of healthy control group.

## Conclusion

Through analysis of stone composition and urine ingredients of urolithiasis, we found some clear relationships between stone components and urinary chemicals. Furthermore, we also discovered that decreased urinary magnesium and urinary oxalic acid were independent risk factors for stones containing calcium oxalate, which were in agreement with previous results. Therefore, we presumed that the urinalysis could be used to predict the recurrence risk for urolithiasis who had calcium oxalate stones.

## Supplementary Information

Supplementary Tables.
