# Supplementary material for: Study of risk factor of urinary calculi according to the association between stone composition with urine component
Source: Sci Rep. 2021 Apr 22;11:8723. doi: 10.1038/s41598-021-87733-7 (PMC8062512; doi:10.1038/s41598-021-87733-7)
Supplement: Supplementary file 1 — Supplementary Tables. [file 41598_2021_87733_MOESM1_ESM.docx]

**Study of risk factor of urinary calculi according to the association between stone composition with urine component**

Pan Wang^1^, Hongxian Zhang^2^, Jiansuo Zhou^1^, Shangjia Jin^1^, Chong Liu^1^, Boxin Yang^1^, Liyan Cui^1*^

Supplementary Table S1 Logistics analysis results of case group

and normal control group

| Variate | *B* | *Wald* | *P* | *OR* | 95%*CI* |
| --- | --- | --- | --- | --- | --- |
| UUA | -0.001 | 2.856 | 0.091 | 1.001 | 1.000-1.001 |
| UP | 0.017 | 0.363 | 0.547 | 0.983 | 0.930-1.039 |
| UMg | -1.095 | 13.673 | 0.000 | 2.990 | 1.673-5.342 |
| UCA | -0.023 | 0.019 | 0.891 | 1.024 | 0.732-1.431 |
| UOXA | -0.035 | 3.828 | 0.050 | 1.035 | 1.000-1.072 |
| UCIT | 0.043 | 0.952 | 0.329 | 0.958 | 0.879-1.044 |
| Constant term | 5.483 | 0.804 | 46.512 |  |  |

Supplementary Table S2 Logistics analysis results of single COX group

and normal control group

| Variate | *B* | *Wald* | *P* | *OR* | 95%*CI* |
| --- | --- | --- | --- | --- | --- |
| UUA | -0.001 | 2.494 | 0.114 | 1.001 | 1.000-1.001 |
| UP | 0.027 | 0.729 | 0.393 | 0.974 | 0.916-1.035 |
| UMg | -1.214 | 11.362 | 0.001 | 3.366 | 1.662-6.818 |
| UCA | 0.085 | 0.187 | 0.666 | 0.918 | 0.623-1.353 |
| UOXA | -0.032 | 2.366 | 0.124 | 1.032 | 0.991-1.075 |
| UCIT | 0.058 | 1.324 | 0.250 | 0.943 | 0.854-1.042 |
| Constant term | -0.804 | 34.231 | 0 |  |  |

Supplementary Table S3 Logistics analysis results of mixed COX group

and normal control group

| Variate | *B* | *Wald* | *P* | *OR* | 95%*CI* |
| --- | --- | --- | --- | --- | --- |
| UUA | -0.001 | 3.156 | 0.076 | 1.001 | 1.000-1.002 |
| UP | 0.004 | 0.010 | 0.919 | 0.996 | 0.915-1.083 |
| UMg | -1.521 | 10.394 | 0.001 | 4.575 | 1.815-11.53 |
| UCA | 0.007 | 0.001 | 0.975 | 0.993 | 0.634-1.554 |
| UOXA | -0.08 | 5.533 | 0.019 | 1.083 | 1.013-1.157 |
| UCIT | 0.059 | 0.62 | 0.431 | 0.943 | 0.815-1.091 |
| Constant term | 6.261 | 20.506 | 0 |  |  |

Supplementary Table S4 Logistics analysis results of other group

and normal control group

| Variate | *B* | *Wald* | *P* | *OR* | 95%*CI* |
| --- | --- | --- | --- | --- | --- |
| UUA | -0.001 | 3.183 | 0.074 | 1.001 | 1.000-1.003 |
| UP | -0.091 | 1.237 | 0.266 | 1.095 | 0.933-1.284 |
| UMg | -0.576 | 1.383 | 0.240 | 1.779 | 0.681-4.644 |
| UCA | -0.381 | 0.971 | 0.325 | 1.464 | 0.686-3.124 |
| UOXA | -0.009 | 0.066 | 0.797 | 1.009 | 0.942-1.081 |
| UCIT | 0.017 | 0.071 | 0.790 | 0.983 | 0.869-1.113 |
| Constant term | 4.948 | 8.61 | 0.003 |  |  |
